# HNF1B-MODY in the Norwegian MODY Registry and the Norwegian Childhood Diabetes Registry: Clinical Insights and Prevalence Informed by Genetic and Functional Evaluation

**DOI:** 10.3390/ijms27115067

**Published:** 2026-06-03

**Authors:** Aishwarya Pavithram, Bente B. Johansson, Erling Tjora, Pernille Svalastoga, Khadra A. Mohamed, Ingvild L. Koløen, Maren Toftdahl, Torild Skrivarhaug, Marc Vaudel, Lise Bjørkhaug, Kristin A. Maloney, Toni I. Pollin, Stefan Johansson, Christine Bellanné-Chantelot, Jørn V. Sagen, Janne Molnes, Pål R. Njølstad

**Affiliations:** 1 Mohn Center for Diabetes Precision Medicine, Department of Clinical Science, University of Bergen, 5021 Bergen, Norway; aishwarya.pavithram@uib.no (A.P.); erling.tjora@helse-bergen.no (E.T.); pernilles@gmail.com (P.S.); khadra.mohamed@ru.nl (K.A.M.); ingvildkoloen@hotmail.com (I.L.K.); maren.toftdahl@gmail.com (M.T.); marc.vaudel@uib.no (M.V.); stefan.johansson@uib.no (S.J.); jorn.v.sagen@ahus.no (J.V.S.); janne.molnes@uib.no (J.M.); 2 Department of Pediatric and Adolescent Medicine, Haukeland University Hospital, 5021 Bergen, Norway; 3 Department of Pediatrics, Oslo University Hospital, 0450 Oslo, Norway; torild.skrivarhaug@medisin.uio.no; 4 Institute of Clinical Medicine, University of Oslo, 0372 Oslo, Norway; 5 Computational Biology Unit, Department of Informatics, University of Bergen, 5020 Bergen, Norway; 6 Department of Genetics and Bioinformatics, Norwegian Institute of Public Health, 5009 Bergen, Norway; 7 Department of Safety, Chemistry, and Biomedical Laboratory Sciences, Western Norway University of Applied Sciences, 5020 Bergen, Norway; lise.bjorkhaug.gundersen@hvl.no; 8 Department of Medicine, University of Maryland School of Medicine, Baltimore, MD 21201, USA; kmaloney1@som.umaryland.edu (K.A.M.); tpollin@som.umaryland.edu (T.I.P.); 9 Department of Medical Genetics, Pitié-Salpêtrière Hospital, Assistance Publique-Hôpitaux de Paris (AP-HP) Sorbonne University, 75013 Paris, France; christine.bellanne-chantelot@aphp.fr; 10 Department of Medical Biochemistry and Pharmacology, Haukeland University Hospital, 5021 Bergen, Norway; 11 Division of Diagnostics and Technology, Akershus University Hospital, 1478 Lørenskog, Norway; 12 Faculty of Medicine, Institute of Clinical Medicine, University of Oslo, 0318 Oslo, Norway; 13 Department of Medical Genetics, Haukeland University Hospital, 5021 Bergen, Norway

**Keywords:** Hepatocyte Nuclear Factor-1B, 17q12 deletion, *HNF1B*-associated disease, monogenic diabetes, MODY, renal structural disease, diabetes registries, functional analyses, VUS, variant interpretation

## Abstract

Interpreting *HNF1B* variants is challenging in clinical practice. We aimed to integrate functional, clinical, and family data to improve variant classification, describe clinical features of carriers and report registry-level prevalence of *HNF1B* alterations. Clinical, genetic, and family data were analyzed from the Norwegian MODY Registry (NMR) and the Norwegian Childhood Diabetes Registry (NCDR). Clinical features of sequence variant and 17q12 deletion (17q12del) carriers were summarized, and variants were classified using ACMG-AMP-ClinGen criteria. Registry-level prevalence was reported with 95% confidence intervals. *HNF1B* sequence variants were functionally assessed, showing that lower transactivation (TA) was associated with higher clinical severity. Eleven variants demonstrated impaired functional activity, with TA inversely correlated with clinical burden (ρ = −0.701, *p* = 0.002). We identified 28 individuals with 17q12del (21 in NMR, seven in NCDR) and 15 individuals carrying 14 unique pathogenic/likely pathogenic (P/LP) sequence variants, all detected in the NMR. Overall, 36/486 probands (7.4%) with genetically confirmed monogenic diabetes in the NMR carried a P/LP *HNF1B* sequence variant or 17q12del. In the NCDR, ~0.2% carried 17q12del (7/3583; 3/7 GADA/IA-2A-positive). Functional data enabled reclassification of three variants. Since many pediatric 17q12del carriers in the NMR were referred for testing due to structural renal anomalies without diabetes, *HNF1B* screening should be considered in children with renal/extra-renal features, irrespective of diabetes or autoantibody status.

## 1. Introduction

Maturity-onset diabetes of the young (MODY) is a form of autosomal-dominant monogenic diabetes with early onset and clinical overlap with type 1 and type 2 diabetes [1,2,3]. MODY is caused by single-gene defects mostly affecting beta-cell development and function, with at least ten known causative genes [4]. Population studies have shown that pathogenic alterations in hepatocyte nuclear factor 1 alpha (*HNF1A*) and glucokinase (*GCK*) account for the largest proportion of MODY (~30–50%), followed by *HNF4A* (~10%) and *HNF1B* (~5%) [5]. In Norway, according to the Norwegian MODY Registry (NMR), HNF1A- and GCK-MODY are most prevalent, followed by HNF1B- and HNF4A-MODY (NMR, unpublished results [J.M and P.R.N.]). Among established MODY subtypes, HNF1B-MODY is distinctive for its multisystemic presentation, combining diabetes with renal and genitourinary structural anomalies, hepatobiliary involvement, uric acid and electrolyte disorders, neurodevelopmental features, and pancreatic hypoplasia [6,7,8,9,10]. The mechanisms underlying HNF1B-MODY primarily involve reduced gene dosage or functional impairment of the protein. Whole-gene deletions (17q12del), encompassing one complete copy of the *HNF1B* gene, are well-established causes of haploinsufficiency [6,11], whereas sequence variants may exert variant-specific effects depending on their type and location. Protein truncating variants (PTVs; e.g., nonsense, frameshift, or canonical splice-site variants) are generally expected to result in loss-of-function (LoF), often through mechanisms such as nonsense-mediated decay (NMD) [12]. In contrast, *HNF1* missense variants affecting functional domains (dimerization domain (DD), DNA-binding domain (DBD), and transactivation domain (TAD)) exhibit variable functional consequences ranging from mild to severe, with or without detectable loss in expression [13,14]. Functional impairments may arise through mechanisms such as impaired dimerization, altered subcellular localization, or reduced DNA binding, ultimately affecting transactivation capacity [15,16].

Despite clear gene-disease association, the interpretation of *HNF1B* missense variants remains difficult. Phenotypic heterogeneity blurs genotype–phenotype correlations [17,18], the high rate of de novo occurrences decreases the ability to perform segregation analyses [19,20], and functional data for missense variants are limited. Moreover, incomplete penetrance and variable expressivity of associated manifestations complicate the interpretation [17,21,22,23,24,25]. Together, these factors complicate variant classification, as different lines of evidence (clinical, segregation, in silico, and functional) may be limited, conflicting, or inconsistently weighted across laboratories. This increases the risk of misclassification, potentially resulting in missed or delayed diagnoses and impacting clinical management. Although efforts are underway to develop *HNF1B*-specific variant interpretation guidelines, current reliance on general ACMG-AMP criteria [26] or adaptation of *HNF1A*-specific guidelines (clinicalgenome.org, accessed 13 May 2026) due to sequence similarity may contribute to inconsistent application of evidence and inter-laboratory variability in classification.

Therefore, the present study focuses on clinical phenotyping of both sequence variants and 17q12del cases, with additional functional evaluation for sequence variants. Specifically, we investigated the NMR and the Norwegian Childhood Diabetes Registry (NCDR) to: (i) describe and compare clinical features among sequence variant and 17q12del carriers; and (ii) integrate functional readouts with clinical and family data to improve variant interpretation. By obtaining a precise variant classification, we can not only provide improved treatment and genetic counselling for individuals but also obtain a more accurate estimate of HNF1B-MODY prevalence. Thus, extending our NCDR-based *HNF1A* framework (lower-bound prevalence ~0.34%) [3,13,27] to *HNF1B*, we aim to precisely classify variants using functional analyses to reduce variants of uncertain significance (VUS) burden, and estimate registry-level prevalence of HNF1B-MODY in NMR and NCDR.

## 2. Results

### 2.1. Study Cohort and HNF1B Alterations

**Figure 1:**
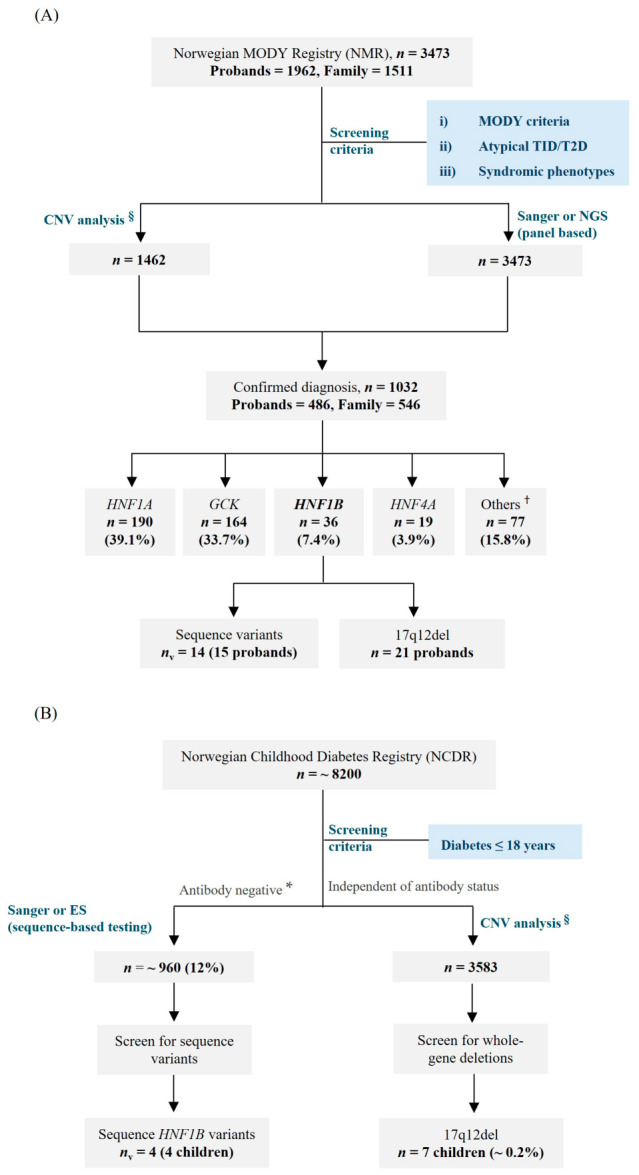
Workflow from referral to genetic diagnosis in the NMR (**A**) and NCDR (**B**). In Panels A and B, “*n*” denotes the number of individuals unless otherwise specified, whereas “*n*_v_” indicates the number of unique sequence variants. Percentages in Panel A were calculated based on the number of probands (*n* = 486) genetically diagnosed with monogenic diabetes. Approximately 88% of individuals in the NCDR were autoantibody-positive at diagnosis and are therefore not routinely screened for monogenic diabetes; however, * 469 age- and sex-matched autoantibody-positive children were included in the genetic analyses as controls, as detailed in [1]. ^†^ Others include less common monogenic diabetes genes; a complete overview of MODY genes is provided in [4]. ^§^ CNV analysis was performed in a subset of individuals from the NMR and NCDR based on DNA availability and study design, as described in [28]. T1D = type 1 diabetes. T2D = type 2 diabetes. CNV = copy-number variant. 17q12del = 17q12 deletion. ES = exome sequencing. NGS = next-generation sequencing.

**Figure 2:**
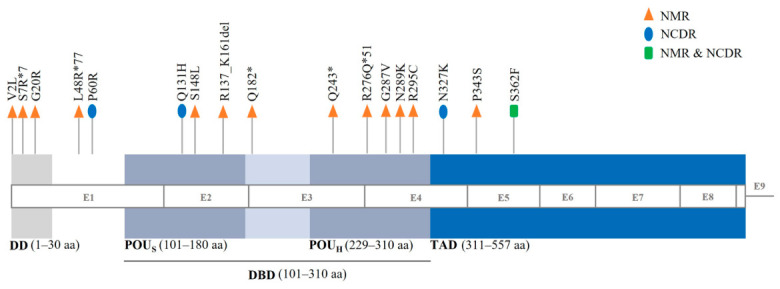
HNF-1B protein domains and sequence variants identified in Norwegian registries. HNF-1B protein consists of an N-terminal dimerization domain (DD), conserved DNA-binding domain (DBD), and a disordered transactivation domain (TAD). Color coding distinguishes different functional domains, and the corresponding size of each domain is described by amino acids (aa), and each variant is labeled according to its respective position. Variants identified in the Norwegian MODY Registry (NMR) are indicated by orange triangles, variants identified in the Norwegian Childhood Diabetes Registry (NCDR) by blue circles, and variants identified in both registries by green squares. * is part of the variant name. E = Exons. aa = amino acids. POU = Pit-1, Oct-1/2, UNC-86. POU_S_ = POU-specific domain. POU_H_ = POU homeodomain.

Among individuals referred for genetic testing in the NMR, 1032 of 3473 (30%) have received a confirmed genetic diagnosis of monogenic diabetes (Figure 1). In this study, across two registries we identified 17 unique *HNF1B* sequence variants among 18 participants (14 in the NMR and four in the NCDR) (Figure 2). One variant (p.S362F) was identified in individuals from both registries. The p.S362F carrier in the NMR cohort developed diabetes at 16 years, while the child in the NCDR cohort was diagnosed with diabetes at the age of 1. First-degree relatives of both individuals were unaffected, and the variant is currently classified as VUS. All pathogenic/likely pathogenic (P/LP) variant carriers were identified in the NMR, whereas all variants identified in individuals from the NCDR were classified as likely benign (LB) or VUS. In addition, 28 individuals (21 probands in the NMR and seven children in the NCDR) were found to carry 17q12del. The majority of 17q12del carriers were identified in the NMR (81%), although one in five were identified through the NCDR (19%). No sex difference was found within and across groups (Table 1). Median age at referral was comparable between P/LP sequence variant carriers and 17q12del carriers (29.0 [IQR 15.5–44.0] vs. 34.0 [IQR 11–41] years; Mann–Whitney U = 589.5, *p* = 0.513).

### 2.2. Clinical Characteristics of P/LP Sequence Variants and 17q12del Carriers

Clinical characteristics of individuals carrying P/LP sequence variants and 17q12del are summarized in Table 1. Renal involvement was universal in both groups, confirming renal disease as the core clinical feature of *HNF1B*-associated disease. Structural renal anomalies were more consistently observed among 17q12del carriers, whereas sequence variant carriers demonstrated greater phenotypic variability. Diabetes was common but not universal in both groups. While three individuals presented with isolated diabetes (e.g., carriers of the p.G20R variant), renal and extra-renal manifestations were frequently observed in the absence of diabetes, underscoring that diabetes is not required for clinical suspicion of *HNF1B*-associated disease.

**Figure 3:**
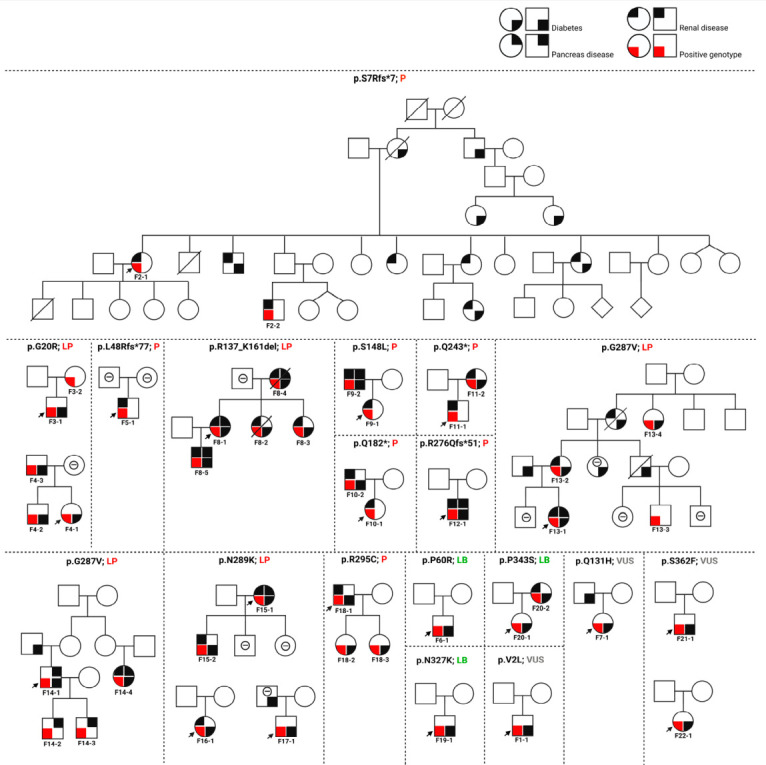
Pedigrees of probands in the NMR carrying *HNF1B* sequence variants. Each panel shows one or more families grouped by the specific sequence variant identified in the proband. Squares and circles represent male and female, respectively. Individuals with unknown sex are represented by diamonds, and deceased individuals are marked by a diagonal line through the symbol. * is part of the variant name. Open symbols with a minus sign (−) indicate individuals who were genetically tested and confirmed negative for the variant. Filled symbols in black represent individuals affected by diabetes (bottom left), renal disease (top left), or pancreatic disease (top right). The proband in each family is indicated with an arrow. Family codes for the proband and other individuals are shown. Individuals positive for the *HNF1B* variant are filled in red (bottom right). Variant classifications were colored red (P/LP), green (LB), and grey (VUS). P/LP = pathogenic/likely pathogenic. LB = likely benign. VUS = variants of uncertain significance.

Beyond diabetes and renal abnormalities, 17q12del patients also presented with neurocognitive impairment and electrolyte disturbances (hypomagnesemia). The apparent absence of these features among P/LP sequence variant carriers and other 17q12del carriers may reflect a lack of systematic screening rather than true absence of the phenotype. Pancreatic, genital tract, and liver abnormalities were also observed in both groups. Overall, clinical burden assessed using the Faguer score [29] was comparable between both 17q12del and P/LP sequence variant carriers. Family pedigrees of probands carrying *HNF1B* sequence variants are shown in Figure 3.

### 2.3. Registry-Level Prevalence of HNF1B Alterations

Seven children in the NCDR were diagnosed with HNF1B-MODY due to 17q12del, corresponding to a prevalence of ~0.2% (7/3583; 95% CI 0.09–0.40%) among those screened, or 1.95 per 1000 children (95% CI 0.95–4.03). When considering the entire NCDR cohort with approximately 8200 children, this may correspond to a lower-bound prevalence estimate of 85 per 100,000 (95% CI 41–176). Of these seven children, three (43%) were positive for pancreatic autoantibodies at diagnosis. One child showed elevated glutamic acid decarboxylase antibodies (GADA; 0.41 antibody index [AI], cut-off < 0.09 AI), while two children showed elevated insulinoma-associated antigen-2 antibodies (IA-2A; >3.00 AI and 0.17 AI, cut-off < 0.11 AI, respectively). All three patients demonstrated a clinical and biochemical phenotype suggesting autoimmune diabetes with no or low insulin production. Among 486 probands in NMR with genetically confirmed monogenic diabetes, HNF1B-MODY accounted for 7.40% (95% CI 5.39–10.08%). When stratified by mutational mechanism, 17q12del was identified in 21/1462 probands (1.43%, 95% CI 0.94–2.18%), while P/LP sequence variants were detected in 15/1962 probands (0.76%, 95% CI 0.46–1.25%), noting that genetic testing strategies differed across individuals and over time, resulting in partially overlapping subsets undergoing CNV and sequence variant analyses. Notably, one 17q12del carrier in the NMR cohort demonstrated clear positivity for all three autoantibodies (GADA 14.4 U/mL; cut-off < 5.0 U/mL, IA-2A 237 U/mL; cut-off < 7.5 U/mL, and zinc transporter 8 antibodies (ZnT8A) 33.6 U/mL; cut-off < 15 U/mL), consistent with an overlap with type 1 diabetes and a diabetes phenotype primarily due to type 1 diabetes, with an additional phenotype attributed to the 17q12del.

### 2.4. Functional Analyses

In this study, the functional impact of variants was examined in full-length HNF-1B using in vitro protein assays. Functionally investigated variants included ten missense (p.V2L, p.G20R, p.P60R, p.Q131H, p.G287V, p.N289K, p.R295C, p.N327K, p.P343S, and p.S362F), three frameshift (p.S7R*7, p.L48R*77, and p.R276Q*51), two nonsense (p.Q182* and p.Q243*), and one in-frame deletion (p.R137_K161del) (Figure 2). To aid interpretation, five variants were incorporated as controls: two well-established pathogenic variants (p.S148L and p.R177* [30]), and two synonymous variants (p.T186= and p.V413=) without predicted splice effects (spliceailookup.broadinstitute.org, accessed 23 February 2026). The p.V413= variant is additionally observed in the population database (gnomAD v4.1.0 Grpmax FAF = 0.006%).

#### 2.4.1. *HNF1B* Gene Variants and Their Classification Prior to Functional Investigations

Among 17 identified sequence variants, ten (p.V2L, p.S7R*7, p.L48R*77, p.P60R, p.Q131H, p.Q243*, p.R276Q*51, p.N327K, p.P343S, and p.S362F) have not been previously reported. Five variants (p.G20R (personal communication and ClinVar VCV000591835.2) [31], p.Q182* [32,33,34,35], p.S148L [22,32,36,37,38,39,40,41], p.G287V (ClinVar VCV000591835.2), p.N289K (personal communication), and p.R295C [17,42,43,44]) were reported by others or listed in ClinVar (www.ncbi.nlm.nih.gov/clinvar, accessed on 23 February 2026). The p.R137_K161del variant was previously reported and functionally characterized by our group [45]. Prior to functional analyses, all variants were classified according to American College of Medical Genetics and Genomics and the Association for Molecular Pathology (ACMG-AMP) guidelines, with specifications from the Clinical Genome Resource (ClinGen) (Table 2). Irrespective of the variant class, all identified variants were included in the functional analyses.

ACMG-AMP-ClinGen guidelines without functional evidence were used to assign an initial classification. Transactivation and DNA-binding activities of variants were presented as % of WT. Functional evidence (e.g., PS3_Supp or BS3_Supp) was added to the classification based on the obtained transactivation data in HeLa cells. Variants with impaired transactivation activity (≤50%) were assigned PS3_Supp, while variants with comparable activity to WT HNF-1B (≥85%) were assigned BS3_Supp, in accordance with the Brnich control criteria [46]. Variants that were reclassified after functional analyses are highlighted with an arrow. POU = Pit-1, Oct-1/2, UNC-86. Supp = Supporting. VUS = variants of uncertain significance.

#### 2.4.2. Effect of *HNF1B* Variants on Transactivation Activity

**Figure 4:**
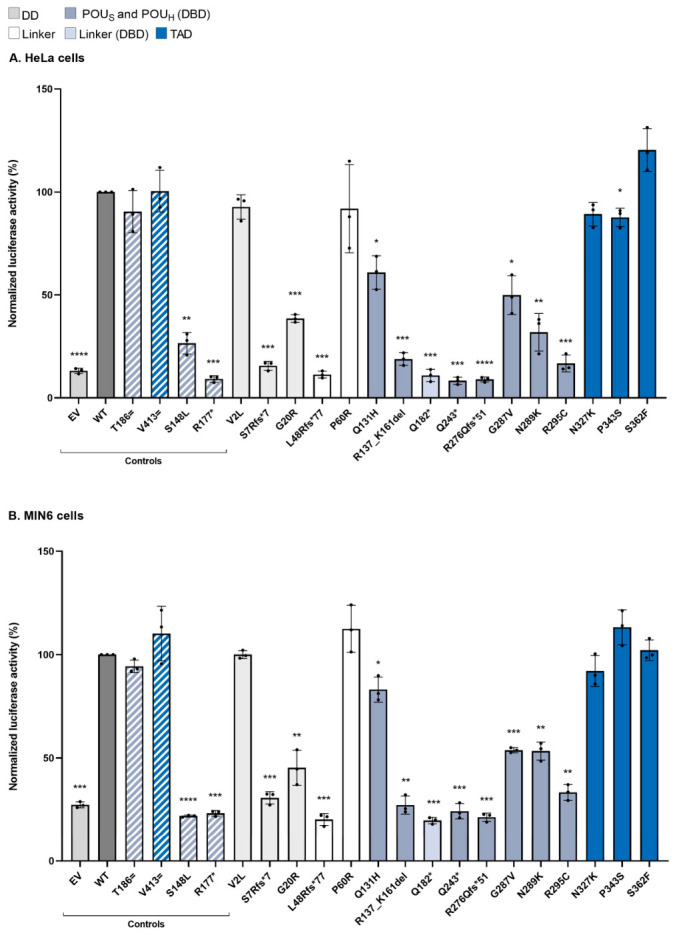
Transactivation profile of HNF-1B protein variants in HeLa (**A**) and MIN6 (**B**) cells. Variants located in the dimerization domain (DD) and DNA-binding domain (DBD) showed significantly reduced transactivation activity, whereas variants in the linker and transactivation domains (TAD) retained activity comparable to WT. Data are expressed as percentage of WT activity (set to 100%). Transactivation activities were normalized to WT HNF-1B measured within the same experimental batch, with WT set to 100%. WT HNF-1B is shown as a solid grey bar. Patterned bars represent the control variants (p.T186=, p.V413=, p.S148L, and p.R177*). Bars represent mean of three biological replicates (*n* = 3), with individual data points shown; error bars indicate standard deviations. Statistical significance is indicated by asterisks (* *p* ≤ 0.05; ** *p* ≤ 0.01; *** *p* ≤ 0.001; **** *p* ≤ 0.0001). EV = empty vector; WT = wild type.

Transactivation potential of *HNF1B* variants examined in lysates of transfected HeLa and MIN6 cell lines revealed similar profiles across both cell lines (Figure 4). All PTVs (p.S7R*7, p.L48R*77, p.Q182*, p.Q243*, and p.R276Q*51) and the deletion variant (p.R137_K161del) showed markedly reduced activity (≤20% in HeLa and ≤30% in MIN6) similar to HNF1B-MODY controls. Four missense variants (p.G20R, p.G287V, p.N289K, and p.R295C) located in either DD or DBD, also showed significantly impaired transactivation potential (≤50% in HeLa cells and ≤55% in MIN6 cells). The p.Q131H variant demonstrated moderately reduced activity (60% in HeLa cells and 80% in MIN6 cells). All other variants demonstrated transcriptional activity comparable to WT HNF-1B (Figure 4).

#### 2.4.3. Nuclear Import and DNA-Binding Analysis of *HNF1B* Variants

**Figure 5:**
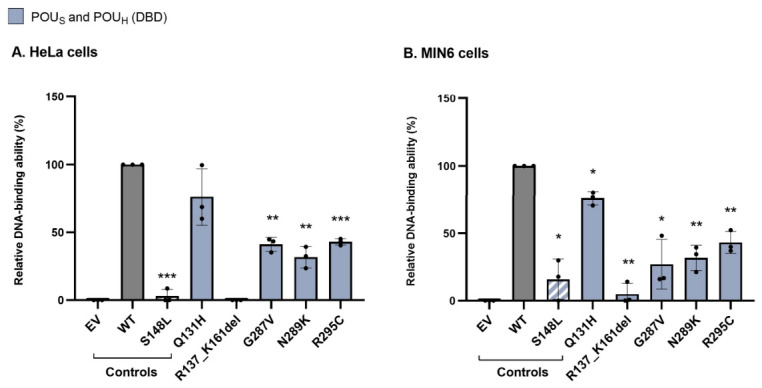
DNA binding capabilities of HNF-1B protein variants from transfected HeLa (**A**) and MIN6 (**B**) cells. Variants p.G287V, p.N289K, and p.R295C showed reduced DNA binding, whereas p.Q131H showed only a modest reduction. The deletion variant p.R137_K161del showed minimal to no detectable DNA binding. Data are expressed as percentage of WT activity (set to 100%). WT HNF-1B is shown as a solid grey bar. Patterned bars represent control variant, p.S148L. Bars represent the mean of three biological replicates (*n* = 3), with individual data points shown; error bars indicate standard deviations. DNA-binding activities were normalized to WT HNF-1B measured within the same experimental batch, with WT set to 100%. Statistical significance is indicated by asterisks (* *p* ≤ 0.05; ** *p* ≤ 0.01; *** *p* ≤ 0.001). EV = empty vector, WT = wild type.

The deletion variant and missense variants in the DBD with impaired transactivation activity were evaluated for their ability to bind to the rat albumin (RA) promoter using electrophoretic mobility shift assays (EMSA). The p.G20R variant, despite reduced transactivation activity, was not included due to its location outside the DBD and markedly reduced nuclear protein expression (Supplementary Figure S1), which precluded reliable assessment of DNA-binding capacity. Variants with near-normal transactivation (≥85% of WT) are expected to retain DNA-binding capacity and were therefore not prioritized for further analysis. In addition, PTVs were also excluded from this analysis due to the expected NMD and/or loss of one or more protein domains (DD, DBD and/or TAD) in the unlikely event of NMD-escape. Among the assessed missense variants, p.G287V, p.N289K, and p.R295C showed markedly impaired DNA binding (≤45% of WT activity) in both cell lines. In contrast, p.Q131H showed only a modest reduction (~75% of WT activity). On the other hand, the deletion variant p.R137_K161del showed minimal to no DNA binding in EMSA, likely due to markedly reduced protein expression in combination with impaired DNA-binding capacity (Figure 5 and Figure S1).

### 2.5. Functional and Clinical Correlations

**Figure 6:**
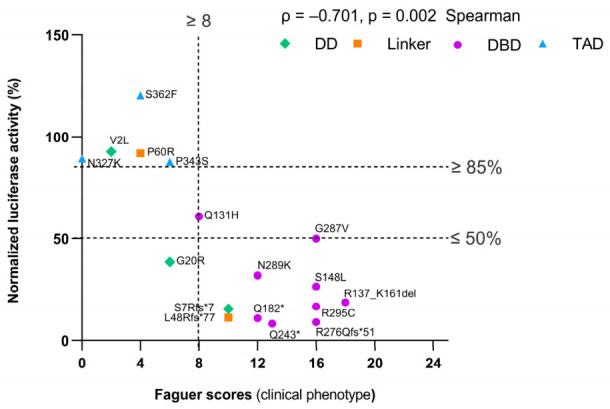
Correlation between transactivation activity and Faguer scores. Transactivation capacity of variants assessed in HeLa cells (expressed as a percentage relative to wild type, 100%) is plotted on the *y*-axis, while the highest Faguer scores obtained for each variant, representing clinical phenotype severity, are shown on the *x*-axis. Each data point represents a variant colored by its protein domain: green for dimerization domain (DD; residues 1–30), orange for linker region (31–100), purple for DNA-binding domain (DBD; 101–310), and blue for transactivation domain (TAD; 311–557). The vertical dashed line represents a Faguer score of 8 set as a threshold for applying the PP4 criteria. The horizontal lines at 50 and 85% were included as a visual reference for impaired and normal transactivation activity, respectively. * is part of the variant name.

To assess concordance between functional activity and clinical severity, we analyzed transactivation activity versus Faguer score across 17 variants. Spearman’s rank correlation showed significant inverse association (ρ = −0.701, *p* = 0.002; 95% CI −0.887 to −0.318), indicating that lower transactivation levels corresponded to greater clinical severity. Except for p.G20R, all variants with impaired transactivation activity exhibited Faguer scores of 8 or higher (Figure 6).

To further evaluate the clinical relevance of transactivation activity, variants were divided into two groups based on activity, ≤50% vs. >50% of WT. Variants with ≤50% transactivation (*n* = 11) had a significantly higher median Faguer score (median = 13.0, interquartile range [IQR] = 11–16), compared to variants in the >50% activity group (*n* = 6), with a median score of 4.0 (IQR = 2–6). The difference was statistically significant (U = 1.5, Z = −3.193, *p* < 0.001). The mean Faguer score in the ≤50% group was 13 ± 3.6, compared to 4.0 ± 2.8 in the >50% group. These findings underscore that the PTVs and DD/DBD missense variants with ≤50% transactivation activity are functionally deleterious and are associated with renal anomalies and/or extra-renal features, while missenses in the linker/TAD regions (e.g., p.V2L, p.P60R, p.N327K, p.P343S, and p.S362F) retained normal HNF-1B function with no specific *HNF1B*-associated abnormalities. There were no significant differences in Faguer score severity between PTVs and damaging missense variant carriers, although this may partly be due to the small sample size.

## 3. Discussion

In this study, we have addressed gaps in *HNF1B* variant interpretation by functionally testing variants identified in patients from two Norwegian diabetes registries. While in vitro assays cannot capture all pathogenic mechanisms, functional results generated using our cDNA system aligned with the clinical phenotype (Faguer scores) of carriers, supporting causality of 11 P/LP variants. Functional data enabled reclassification of three variants (p.P60R, p.N327K, and p.P343S) to LB and provided data to reduce the likelihood of pathogenicity of two VUS (p.V2L and p.S362F). Conversely, impaired transactivation activity observed for variants initially classified as VUS (p.G20R, p.G287V, and p.N289K) prompted targeted clinical and segregation follow-up, which subsequently provided additional evidence for reclassification to LP. These findings are consistent with previous *HNF1B* studies showing that P/LP variants typically impair transactivation activity [47], whereas variants with near-normal activity are less likely to be disease-causing [48]. Importantly, our results highlight the value of functional assays not only in supporting variant classification but also in guiding targeted clinical follow-up.

Across cohorts, *HNF1B* accounted for 7.4% of monogenic diabetes cases in the NMR (among 486 probands with genetically confirmed MODY) and ~0.2% in the NCDR (among 3583 individuals screened). These estimates are derived from registry-based cohorts and are influenced by differences in patient ascertainment and genetic testing strategies. In the NMR, individuals are referred for genetic testing based on clinical suspicion of monogenic diabetes. In the NCDR, panel/exome testing is primarily performed in autoantibody-negative individuals, and CNV analysis was not systematically performed in all individuals in either registry. Consequently, some HNF1B-MODY cases, including 17q12del cases, may have been missed, particularly since autoantibody positivity does not fully exclude HNF1B-MODY. Therefore, these estimates should be interpreted as lower-bound estimates rather than true population prevalence and are not directly comparable across registries or mutation classes.

In total, across both registries, 26 children aged 0–18 years at the time of diagnosis were identified as having HNF1B-MODY. Within the NMR cohort, pediatric carriers of the 17q12del were primarily ascertained based on structural renal anomalies rather than the clinical presentation of diabetes. Notably, autoantibody positivity did not exclude HNF1B-MODY in our registries, as four individuals with 17q12del were positive for one or more islet autoantibodies (GADA, IA-2A, ZnT8A). These findings highlight that HNF1B-MODY can overlap clinically with type 1 diabetes and that additional syndromic features should be considered to support accurate diagnosis. Particularly, two of these autoantibody-positive individuals with 17q12del exhibited features atypical for classical type 1 diabetes, including hypomagnesemia in one individual and early-onset renal dysplasia (at two weeks of age) in another. However, limited clinical data for the remaining two individuals restricted detailed phenotypic comparison. Clinically, these findings support considering *HNF1B* genetic testing in children with renal or pancreatic structural abnormalities and/or electrolyte disturbances, irrespective of diabetes status or single autoantibody positivity. This may reduce missed diagnoses and enable earlier detection and management of associated comorbidities.

Variants with pathogenic/likely pathogenic effects

The p.G20R variant was identified in two unrelated families within the NMR. In the F4 pedigree, the proband, a sibling, and their father carried the variant; all developed diabetes, and the father had gout, a feature compatible with HNF1B-MODY [49]. Recurrence of the variant across multiple unrelated families (ClinVar VCV000591835.2; accessed January 19, 2026), its segregation through three meioses [31] and the markedly reduced transactivation activity together support a LP classification (Figure 4). Notably, none of the p.G20R carriers in our cohort had known renal disease or structural renal abnormalities at diagnosis, underscoring the need for clinical surveillance in families (Table 2).

Four missense variants in the DBD (p.S148L, p.G287V, p.N289K, and p.R295C) were classified as P/LP. All these variant carriers reported renal, pancreatic anomalies and/or diabetes. In family F18, the proband harboring the p.R295C variant has renal failure. Moreover, this residue has repeatedly been linked to renal and extra-renal involvement, warranting close surveillance of the family members in our cohort [17,37,43]. Furthermore, alternative variants of N289 and R295 residues (e.g., p.N289D (unpublished data), p.R295P, p.R295H) have also been reported to cause HNF1B-MODY [22,36,37]. The p.R137_K161del (24 aa deletion) classified as LP was identified in five related individuals, all with diabetes, renal and extra-renal features [45].

Three frameshift (p.S7R*7, p.L48R*77, and p.R276Q*51) and two nonsense (p.Q182* and p.Q243*) variants were classified as P/LP according to ACMG-AMP and demonstrated consistent LoF in our cDNA-based assays (Figure 4). Based on the position of the premature stop codons, these variants are predicted to trigger NMD of the mRNA transcript; however, even in the unlikely event that they escape NMD, they are still expected to produce truncated proteins lacking key functional domains. Because the expression plasmids employed in the functional assays lack intronic sequences, transcripts of these PTVs may escape NMD in vitro. As a result, these assays do not recapitulate the physiological mRNA degradation process but instead assess the residual activity of any truncated protein that is produced.

While functional assays provide strong evidence for variant classification, the relatively small number of individuals carrying P/LP missense variants limits the strength of the genotype–phenotype correlations. In the current study, variants are distributed across all protein domains; however, functionally impaired missense variants are predominantly observed in the DD and DBD, whereas variants in the linker and TAD appeared relatively tolerant to the missense substitutions studied here. Consequently, the number of variants that are both functionally impaired and clinically well-characterized remains limited. Consistent with this, although many individuals carrying P/LP variants exhibit high Faguer scores, the relationship between functional impairment and clinical severity is not absolute. For example, although the p.G20R variant demonstrated impaired transactivation, carriers in our cohort exhibited relatively mild clinical features and did not fulfill PP4 criteria. We further acknowledge that the inverse correlation between transactivation activity and clinical severity may be influenced by several confounding factors. Clinical expression of *HNF1B*-associated disease is highly variable, with both intra- and inter-familial heterogeneity observed. Carriers of the same variant differed in age at onset and organ involvement. In addition, younger individuals (e.g., p.Q182* and p.G287V carriers) had not yet developed diabetes, suggesting age-dependent penetrance. Ascertainment bias may also contribute, as individuals with multisystem involvement are more likely to be referred for genetic testing, whereas milder cases may have been missed, especially prior to 2020. In addition, genetic and environmental modifiers may influence clinical severity. Taken together, these findings highlight the heterogeneity of *HNF1B*-related disease, and larger cohorts will be required to more reliably define genotype-phenotype relationships.

Although in vitro assays provide a robust and scalable first-line approach for functional assessment, cDNA-based overexpression systems used in HeLa and MIN6 cells do not fully recapitulate endogenous *HNF1B* regulation in disease-relevant tissues and may not capture tissue-specific effects or physiological expression levels. Further studies in more physiologically relevant models will be important to improve our understanding of variant effects in endogenous contexts.

Likely benign variants

Functional and clinical evidence supported reclassification of p.P60R, p.N327K, and p.P343S variants to LB (Table 2). Population frequency data met BS1 for p.P60R (Grpmax FAF = 0.0083%) and p.N327K (Grpmax FAF = 0.0055%; gnomad.broadinstitute.org; accessed 19 January 2026) according to *HNF1A* frequency thresholds (clinicalgenome.org/affiliation/50016). Phenotypically, the p.P60R carrier was positive for GADA (0.12 AI; cut-off < 0.09 AI). The p.N327K carrier was positive for both GADA (1.54 AI; cut-off < 0.09 AI) and IA-2A (0.38 AI; cut-off < 0.11 AI) and with markedly reduced C-peptide levels (<50 pmol/L; reference 220–1400 pmol/L), consistent with autoimmune type 1 diabetes. The p.P343S proband was obese (BMI 37.9 kg/m^2^) with high C-peptide levels (2360 pmol/L), suggestive of type 2 diabetes/insulin resistance. A family history of diabetes was absent for p.P60R and p.N327K variant carriers; for p.P343S, the mother was also a carrier, but available data did not demonstrate a segregating *HNF1B* phenotype.

Variants with uncertain significance

Two variants (p.V2L and p.S362F) remained VUS (Table 2) after functional evaluation. Both showed normal transactivation (≥85%) in HeLa and MIN6 cells. Clinically, carriers had diabetes without extra-pancreatic features. The p.V2L carrier was diagnosed at 40 years of age with elevated C-peptide levels (2220 pmol/L), suggestive of insulin resistance, and the p.S362F proband was obese (BMI 30.1 kg/m^2^). Transactivation activity levels comparable with WT (BS3_Supporting) for these variants and the absence of *HNF1B*-specific features argue against *HNF1B* causality. Alternative etiologies such as type 2 diabetes remain possible. For the last variant, p.Q131H, limited clinical data and modest functional impairment (Figure 4 and Figure 5) provided insufficient evidence to classify it as either B/LB or P/LP; thus, it remains a VUS.

## 4. Materials and Methods

### 4.1. Study Participants and Genetic Testing

The NMR is a nationwide, population-based registry established in 1997 [38]. The registry comprises established and suspected cases of monogenic diabetes referred by physicians for genetic testing. By April 2025, NMR comprised 3473 participants (1962 probands and 1511 family members) and the registry was established for diagnostic and research purposes, with the goal of improving diagnosis and treatment of these monogenic disorders. In routine practice, clinicians refer patients based on a clinical suspicion of monogenic diabetes using the following criteria: (1) diabetes in a first-degree relative; (2) onset before ~35 years of age in at least one family member; (3) absence of pancreatic autoantibodies; (4) BMI ≤ 30 kg/m^2^, and (5) atypical presentation of type 1 diabetes (low insulin requirements (<0.5 U/kg/day), no autoantibodies, or an unusual disease course) [38,50]. However, a patient does not necessarily need to meet all criteria for referral and subsequent analysis. Analyses of diabetes-related autoantibodies were performed at two different laboratories (the Hormone Laboratory at Haukeland University Hospital and Aker/Oslo University Hospital), using different methods and laboratory-specific cut-off values. Genomic DNA was extracted using standard procedures. Patients with suspected monogenic diabetes underwent genetic testing: prior to 2020, clinical judgement-directed Sanger sequencing of *HNF1A*, *GCK*, *HNF1B*, *HNF4A*, *INS*, *KCNJ11*, and *ABCC8*; from 2020 onward, panel-based exome sequencing.

The NCDR includes approximately 8200 participants (July 2023) diagnosed with diabetes before 18 years of age since 2002 [3], covering ~99.6% of pediatric cases in Norway. At diagnosis, clinical data, biochemical measures (C-peptide, GADA, IA-2A, and ZnT8A), DNA, and serum are collected. In total, 960 antibody negative individuals (13% of all participants) as well as 469 age- and sex-matched participants with autoimmune diabetes were investigated for variants in monogenic diabetes genes (including *HNF1B*) using panel sequencing or exome sequencing [1,2]. In addition, 1462 individuals included in the NMR and 3583 of the ~8200 individuals included in the NCDR were additionally genotyped using the Illumina Infinium Global Screen Array-24 v2.0 (Illumina, San Diego, CA, USA) to detect whole-gene deletions and Multiplex Ligation-Dependent Probe Amplification (MLPA; P241-E1 MODY, MRC Holland, Amsterdam, The Netherlands) was subsequently used to confirm pathogenic CNVs [28]. However, in the NMR, MLPA-based CNV analysis was implemented routinely in parallel with panel-based testing from 2016 onwards. Prior to 2016, CNV testing was not performed systematically, and approximately 200 probands without a confirmed genetic diagnosis were not assessed for CNVs. Figure 1 presents the stepwise flow chart from referral to genetic testing, and subsequent genetic diagnosis in the NMR/NCDR registries.

### 4.2. Clinical Data

Clinical characteristics of 17q12del and P/LP sequence variant carriers are summarized in Table 1. Continuous variables include age at referral, age at diabetes diagnosis, BMI, and HbA1c. Categorical variables include sex, registry source (NMR/NCDR), diabetes at diagnosis, insulin at referral, and established HNF1B-MODY features.

### 4.3. HNF1B Constructs and Cell Culture

For plasmid construction, variants were introduced into the full-length human *HNF1B* cDNA (NCBI NM_000458.3) within the pcDNA3.1/His B vector via the QuikChange II XL Site-Directed Mutagenesis Kit (Agilent Technologies #200522-5, Santa Clara, CA, USA) utilizing the primer sets detailed in Supplementary Table S2. Plasmids were transformed into ultracompetent E. coli cells (DH5α) and DNA was extracted using the plasmid midi kit (QIAGEN #12243, Hilden, Germany). Introduction of the genetic variant was verified by Sanger sequencing using BigDye Terminator v.3.1 on an ABI 3100 Genetic Analyzer (Applied Biosystems, Foster City, CA, USA). In vitro assessments were performed in HeLa (CCL-2 ATCC, Manassas, VA, USA) and MIN6 cells, received from Prof. R.N. Kulkarni at Joslin Diabetes Center (Harvard Medical School, Boston, MA, USA). While the former is derived from human cervical epithelial tissue, the latter is a pancreatic beta cell line originating from transgenic C57BL/6 mouse insulinoma. Notably, HeLa cells do not express HNF-1B endogenously, whereas MIN6 allows for the investigation of *HNF1B* variants under more physiologically relevant conditions. Both cell types were cultured, maintained, and transiently transfected using Lipofectamine 2000 Transfection Reagent (Invitrogen, Thermo Fisher Scientific #11668-019, Waltham, MA, USA) and Opti-MEM Reduced Serum Medium (Gibco, Thermo Fisher Scientific #31985-062, Waltham, MA, USA). A commercial vector encoding green fluorescent protein (GFP) (Amaxa, Lonza #V4XC-2024, Cologne, Germany) served as a control for transfection efficiency.

### 4.4. Protein Functional Analyses

Functional analyses were performed to assess the transactivation activity, DNA-binding and subcellular localization of variants in HeLa and MIN6 cells as described in our previous study [51].

#### 4.4.1. Transactivation Assay

To assess the transactivation potential of variants, HeLa and MIN6 cells were transiently co-transfected with three plasmids per condition: (i) an effector plasmid encoding either an *HNF1B* variant, WT HNF-1B, or EV; (ii) a firefly luciferase reporter plasmid driven by either the RA promoter (in HeLa cells) or the mouse *GLUT2* promoter (in MIN6 cells); and (iii) an internal control plasmid in which the constitutively active simian virus 40 (SV40) promoter drives *Renilla* luciferase expression. The RA and *GLUT2* promoters contain well-characterized high-affinity HNF-1 binding sites and are widely used in functional studies of HNF-1 transcription factors. These promoters provide robust transactivation signals with low or minimal background, enabling clear discrimination between functionally impaired and normal variants [3,14,51,52,53]. After 24 or 48 h, the cells were harvested, and the luciferase activities measured using the Dual-Luciferase Reporter Assay System (Promega #E1910, Fitchburg, WI, USA) with a Centro XS3 LB 960 microplate luminometer (Berthold Technologies, Bad Wildbad, Germany). The functional effect of each variant was estimated relative to WT HNF-1B activity after normalization of firefly luciferase against *Renilla* luciferase activity.

#### 4.4.2. Subcellular Fractionation

Subcellular fractionation was performed in order to isolate nuclear and cytosolic cell fractions from transiently transfected HeLa and MIN6 cells. In short, pellets from whole-cell lysates were first suspended in a buffer (A) consisting of 10 mM HEPES (pH 7.8), 1.5 mM MgCl_2_, 10 mM KCl, 0.10% (*v*/*v*) IGEPAL, 0.5 mM DTT, and 1 cOmplete Mini EDTA-free Protease Inhibitor Cocktail tablet (Sigma-Aldrich #11836170001, St. Louis, MO, USA), with the total volume adjusted to 10 mL with Milli-Q water. Following incubation and brief centrifugation at 16,250× *g* (4 °C), the supernatants, containing the cytosolic fractions, were collected. Next, the pellets were washed twice in buffer A before thorough resuspension in a second buffer (B) comprising 20 mM HEPES (pH 7.8), 420 mM NaCl, 1.5 mM MgCl_2_, 0.2 mM EDTA, 0.5 mM DTT, and 1 cOmplete Mini EDTA-free Protease Inhibitor Cocktail tablet (Sigma-Aldrich #11836170001), again with the overall volume adjusted to 10 mL with Milli-Q water. The pellets were then vortexed and finally centrifuged for 15 min at 16,250× *g* (4 °C) before harvesting the nuclear fractions.

Prior to SDS-PAGE and immunoblotting, the total amount of protein in each sample was quantified using the Pierce BCA Protein Assay Kit (Thermo Fisher Scientific #23225, Waltham, MA, USA), ensuring an equal loading amount of 5 µg protein from each sample. In addition to anti-HNF-1B antibody (Sigma-Aldrich #HPA002083), antibodies against topoisomerase I (Abcam #ab196642, Cambridge, UK) and topoisomerase II alpha (Cell Signaling Technology #12286S, Danvers, MA, USA) served as nuclear markers for HeLa and MIN6 cells, respectively, and alpha-tubulin (Abcam #ab40742) served as a cytosolic marker in both cell lines. HNF-1B protein levels were quantified by normalizing to topoisomerase I or II alpha to obtain HNF-1B/topoisomerase ratios. These ratios were expressed relative to WT and used to adjust the amount of nuclear protein required for EMSA.

#### 4.4.3. DNA-Binding Studies

The DNA-binding abilities of the five variants situated in the DBD of the HNF-1B protein were studied using EMSA. In short, equal protein amounts of nuclear fractions from both HeLa (5 µg) and MIN6 cells (10 µg) transfected with these variants were incubated together with double-stranded cyanine 5 (Cy5)-labeled oligonucleotides containing the RA promoter (5′-TGT GGT TAA TGA TCT ACA GTT A-3′). Optimal binding conditions were facilitated using the Odyssey EMSA Buffer Kit (LI-COR Biosciences #829-07910, Lincoln, NE, USA), and the resulting reactions were separated by gel electrophoresis, visualized with the ChemiDoc MP imaging system (Bio-Rad Laboratories, Hercules, CA, USA), and quantified in terms of signal strength relative to WT HNF-1B.

### 4.5. Variant Interpretation

*HNF1B*-specific variant interpretation guidelines have so far not been published by the ClinGen Monogenic Diabetes Expert Panel (MDEP). Thus, all variants were classified according to MDEP *HNF1A*-specific ACMG-AMP guidelines (clinicalgenome.org/affiliation/50016), adapted for the *HNF1B* gene where applicable. The ACMG-AMP framework provides a structured approach to variant classification, categorizing variants as B/LB or P/LP based on weighted evidence [26], with updates made to the general guidelines made by the ClinGen [54]. Benign criteria applied in this study include BS1, BS3_Supporting, BP4, and BP7 while pathogenic criteria comprise PVS1, PS4/PS4_Moderate, PM1/PM1_Supporting, PM4_Moderate, PS2/PM6_Supporting, PS2/PM6_Moderate, PS2/PM6_Very strong, PP1/PP1_Moderate/PP1_Strong, PP3, PP4, PM2_Supporting, and PS3_Supporting. For individual criterion details and cut-offs, refer to Supplementary Table S3. The transactivation thresholds used for PS3_Supporting (≤50% of WT activity) and BS3_Supporting (≥85% of WT activity) were empirically calibrated using benchmark P/LP and B/LB control variants included in the transactivation assay (unpublished data), in accordance with the Brnich et al. recommendations for functional evidence application [46]. AlphaMissense was used as an in silico prediction tool to support application of the BP4 and PP3 criteria [55]. Clinical features were scored according to the Faguer et al. specifications (PP4) [29]. PP4 criterion was applied if at least one of the variant carriers had a score of 8 (pre-specified threshold) or higher.

### 4.6. Statistics

Statistical analyses were performed using SPSS (version 29.0.2.0). Results are presented as mean ± standard deviation or as median with interquartile ranges, as appropriate. Continuous variables were summarized as median (25–75th percentiles), and categorical variables as counts and percentages (%). Non-parametric correlations were assessed using Spearman’s rank correlation coefficient (ρ), and group comparisons of medians were performed using the Mann–Whitney U test. For functional assays (transactivation and DNA-binding), statistical significance was evaluated using two-tailed unpaired Student’s *t*-tests with unequal variances (Welch’s *t*-test), comparing each variant to WT (set as 100%). Both transactivation and DNA-binding assays were performed in three independent biological replicates (*n* = 3), and individual data points represent these replicates. No multiple-testing correction was applied, as each variant was evaluated independently against WT to assess variant-specific functional effects. A *p*-value < 0.05 was considered statistically significant. Registry-level prevalence was expressed as proportions with two-sided 95% confidence intervals (CIs) calculated using the Wilson method. Data were plotted using GraphPad Prism 9 (version 9.5.1, GraphPad Software, San Diego, CA, USA).

## 5. Conclusions

Incorporating functional assays with clinical and family data improved *HNF1B* variant interpretation and reduced diagnostic uncertainty in these registries. *HNF1B* accounted for a substantial proportion of MODY in our cohorts, with many cases identified through renal and extra-pancreatic manifestations rather than diabetes alone. Autoantibody positivity did not exclude HNF1B-MODY in children, highlighting limitations of classical diagnostic criteria. Notably, reliance on classical MODY criteria alone would have resulted in missed diagnoses, underscoring the need for broader screening strategies. Together, these findings support the integration of functional data into clinical diagnostics and continue to recommend *HNF1B* genetic testing in individuals with suggestive renal or multisystem features, irrespective of diabetes or islet autoantibody status. Early molecular diagnosis enables appropriate surveillance of renal and systemic complications, informs treatment decisions, and improves genetic counselling and long-term clinical management.

## Figures and Tables

**Table 1:** Summarized clinical characteristics of all P/LP sequence variants and 17q12del carriers.

Characteristics	Total	P/LP Variant Carriers	Total	17q12del Carriers
N (all carriers)	—	35		37
Female (%)	35	18 (51)	37	20 (54)
NMR (%)	35	35 (100)	37	30 (81)
NCDR (%)	—	—	37	7 (19)
Age at referral, years (IQR)	35	29.0 (15.5–44.0)	37	34 (11–41)
Diabetes present (%)	34	25 (74)	37	25 (68)
DM/renal disease in first degree relatives (%)	31	29 (94)	22	22 (100)
Age at diabetes diagnosis, years (IQR)	21	25 (16.0–36.0)	20	27.5 (17.7–36.2)
BMI at referral, kg/m^2^ (IQR)	19	22.6 (18.8–24.3)	20	22 (21.0–24.4)
HbA1c at referral, % (IQR)	28	6.95 (5.8–8.15)	24	7 (6.1–8.1)
HbA1c, mmol/mol (IQR)	28	52.5 (40–65.5)	24	52.5 (43.2–65.7)
Insulin treatment at referral (%)	28	19 (68)	27	13 (48)
HNF1B-MODY features				
Renal disease (%)	22	22 (100)	18	18 (100)
Electrolyte or uric acid imbalance (%)	3	3 (**100**)	8	8 (100)
Pancreatic structural abnormality or exocrine pancreatic insufficiency (%)	14	11 (78)	4	4 (**100**)
Genital tract abnormality (%)	2	2 (**100**)	2	2 (**100**)
Liver test abnormality, cholestasis (%)	3	3 (**100**)	2	2 (**100**)
Gout and hyperparathyroidism (%)	1	1 (**100**)	—	—
Intellectual/Learning disability (%)	—	—	4	4 (**100**)

Data are presented as median (interquartile range [IQR], 25th—75th percentile) for continuous variables and as *n* (%) for categorical variables. For each characteristic, *n* indicates the number of individuals with available data (denominator shown in the “Total” column). Percentages were calculated using the number with available data as the denominator. Both probands and affected family members carrying P/LP sequence variants or 17q12del were included. Items marked with ** indicate very small denominators (*n* ≤ 4) and should be interpreted with caution. P/LP = pathogenic/likelypathogenic; del = deletion; NMR = Norwegian MODY Registry; NCDR = Norwegian Childhood Diabetes Registry.

**Table 2:** ACMG-AMP variant classification before and after functional analysis.

Variants			ACMG-AMP	Functional Studies in HeLa (% WT)		Additional Evidence	
Amino Acid Change	Nucleotide Change	Variant Position Within Domains	Class (Prior to Functional Analyses)	TA Activity	DNA-Binding Activity	Functional Evidence	New Class
p.V2L	c.4G>C	Dimerization	VUS	92.7		BS3_Supp	VUS
p.S7Rfs*7	c.18del	Dimerization	P	15.5			P ^§^
p.G20R	c.58G>A	Dimerization	LP	38.5		PS3_Supp	LP
p.L48Rfs*77	c.143del	Hinge region	P	11.3			P ^§^
p.P60R	c.179C>G	Hinge region	VUS	91.8		BS3_Supp	VUS -> LB
p.Q131H	c.393A>T	POU-specific	VUS	60.9	76.1		VUS
p.R137_K161del	c.410_484del	POU-specific	LP	18.8		PS3_Supp	LP
p.Q182*	c.544C>T	Linker	P	10.9			P ^§^
p.Q243*	c.727C>T	POU-homeo	P	8.3			P ^§^
p.R276Qfs*51	c.827del	POU-homeo	P	9.01			P ^§^
p.G287V	c.860G>T	POU-homeo	LP	49.9	41.2	PS3_Supp	LP
p.N289K	c.867C>G	POU-homeo	LP	31.9	31.9	PS3_Supp	LP
p.R295C	c.883C>T	POU-homeo	P	16.7	43.1	PS3_Supp	P
p.N327K	c.981C>G	Transactivation	VUS	89.2		BS3_Supp	VUS -> LB
p.P343S	c.1027C>T	Transactivation	VUS	87.6		BS3_Supp	VUS -> LB
p.S362F	c.1085C>T	Transactivation	VUS	120.3		BS3_Supp	VUS
Controls							
p.T186=	c.558A>G	POU-specific	VUS	90.4		BS3_Supp	VUS -> LB
p.V413=	c.1239C>T	Transactivation	LB	100.4		BS3_Supp	LB
p.S148L ^†^	c.443C>T	POU-specific	P	26.5	3.02	PS3_Supp	P
p.R177*	c.529C>T	POU-specific	P	9.2			P ^§^

^§^ As per ACMG-AMP, PVS1 (predicted NMD) is applied and not PS3_supp to avoid overweighting the same LoF mechanism. * is part of the variant name. ^†^ The p.S148L variant was observed in individuals of the NMR cohort, although functionally assessed as part of the controls, being a well-established pathogenic variant. Detailed evidence criteria applied for each variant are listed in the Supplementary Table S1.

## Data Availability

The data used in this study were obtained from Norwegian MODY and Norwegian Childhood Diabetes Registries and contain sensitive patient information. All original contributions presented in this study are included in the article/Supplementary Material. Detailed clinical tables can be provided upon request, and additional inquiries can be directed to the corresponding author.

## Supplementary Materials

The following supporting information can be downloaded at: https://www.mdpi.com/article/10.3390/ijms27115067/s1. References [16,29,46,54] are cited in the Supplementary Materials.

## Abbreviations

The following abbreviations are used in this manuscript:

MODY	Maturity-Onset Diabetes of the Young
NMR	Norwegian MODY Registry
NCDR	Norwegian Childhood Diabetes Registry
HNF-1B	Hepatocyte Nuclear Factor-1B
DD	Dimerization Domain
DBD	DNA-Binding Domain
TAD	Transactivation Domain
NLS	Nuclear Localization Signal
EV	Empty Vector
WT	Wild Type
EMSA	Electrophoretic Mobility Shift Assay
RA	Rat Albumin
GLUT2	Glucose Transporter 2
ACMG-AMP	American College of Medical Genetics and Genomics and the Association for Molecular Pathology
ClinGen	Clinical Genome Resource
MDEP	Monogenic Diabetes Expert Panel
P/LP	Pathogenic/Likely Pathogenic
B/LB	Benign/Likely Benign
VUS	Variant(s) of Uncertain Significance
NMD	Nonsense-Mediated Decay
