# Supplementary material for: HNF1B-MODY in the Norwegian MODY Registry and the Norwegian Childhood Diabetes Registry: Clinical Insights and Prevalence Informed by Genetic and Functional Evaluation
Source: Int J Mol Sci. 2026 Jun 3;27(11):5067. doi: 10.3390/ijms27115067 (PMC13257315; doi:10.3390/ijms27115067)
Supplement: Supplementary file 1 [file ijms-27-05067-s001.zip › ijms-4195879-supplementary.pdf]

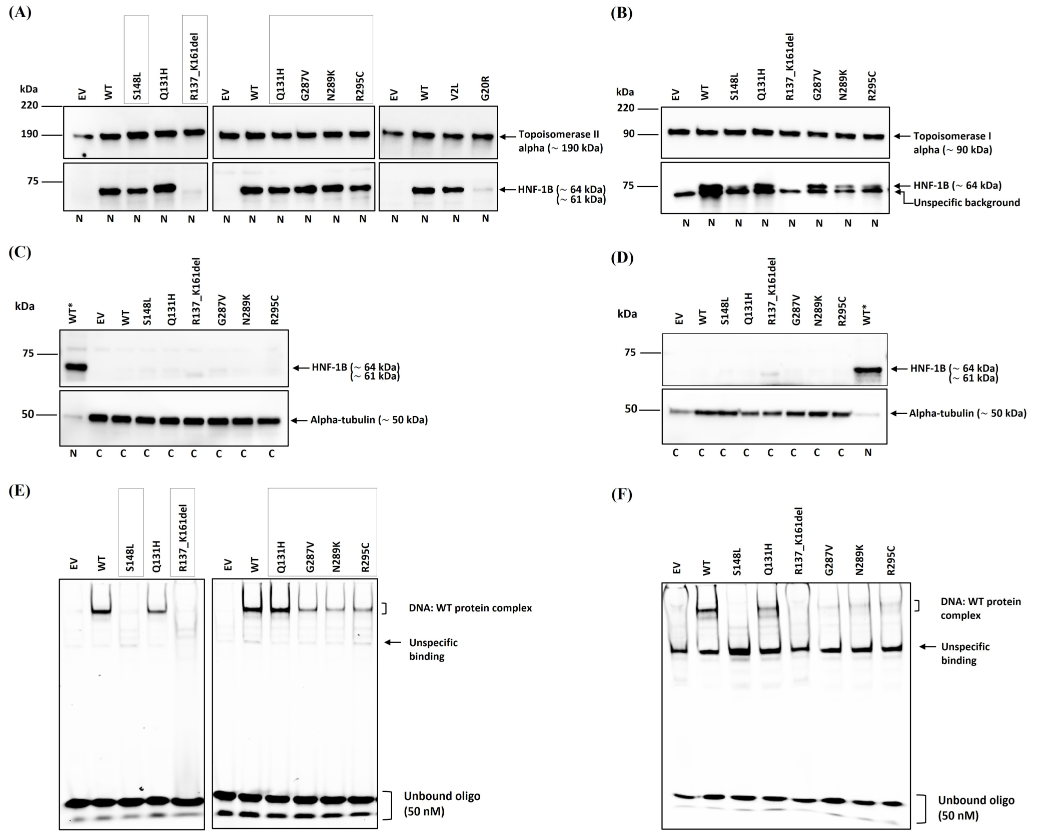

Supplementary Figure S1. DNA binding capacity of HNF-1B protein variants. Cells were transfected with EV, WT, or *HNF1B* variant plasmids and lysed 24- or 48-hour post-transfection in HeLa or MIN6 cells. Equal amounts of protein (5 µg) from cytosol and nuclear fraction were analyzed using SDS-PAGE and western blotting. Full-length HNF-1B was detected at ~64 kDa, while the deletion variant band is expected to migrate at ~61 kDa. WT* indicates a WT nuclear fraction included in the cytosol panel as an antibody control for HNF-1B detection. Representative blots for nuclear (**A**,**B**) and cytosolic (**C**,**D**) fractions are shown for HeLa and MIN6 cells, respectively. Nuclear and cytosol fractions were assessed in three biological replicates ($n = 3$). Equal amounts of nuclear lysates from HeLa (5 µg) and MIN6 (10 µg) cells were incubated with an oligonucleotide containing the consensus RA-binding site together with EMSA components, and the samples were resolved on retardation gels. Representative EMSA blots from HeLa and MIN6 cells are shown in (**E**,**F**). Boxed panels in the HeLa nuclear fractions (A) and EMSA blots (E) indicate lanes used for densitometric quantification and denote their origin (initial versus later reruns performed under identical conditions). For variants p.Q131H, p.G287V, p.N289K, and p.R295C, later reruns were performed with their respective EV and WT controls to reduce inter-blot variability. Quantification for each biological replicate was performed among samples run on the same gel/blot and normalized to the WT control included on that blot. EMSA runs were performed in three biological replicates in both cell lines ($n = 3$). EV = Empty vector. WT = wild type.

Supplementary Table S1. Detailed ACMG-AMP evidence applied for *HNF1B* variants.

Amino Acid Change	Nucleotide Change	Combined ACMG-AMP Evidence	ACMG-AMP Class
p.V2L	c.4G>C	PM1_Supp, PM2_Supp, BS3_Supp	VUS
p.S7Rfs*7	c.18del	PVS1, PP1, PM2_Supp, PP4	P §
p.G20R	c.58G>A	PM1_Supp, PP3, PM2_Supp, PP1_Mod, PS4_Mod, PS3_Supp	LP
p.L48Rfs*77	c.143del	PVS1, PM2_Supp, PS2/PM6_Mod, PP4	P §
p.P60R	c.179C>G	BS1, BS3_Supp	VUS -> LB
p.Q131H	c.393A>T	PM1_Supp, PM2_Supp, PP3	VUS
p.R137_K161del	c.410_484del	PM2_Supp, PM4_Mod, PP1_Mod, PP4, PM1_Mod, PS3_Supp	LP
p.Q182*	c.544C>T	PVS1, PS4_Mod, PM2_Supp, PP4	P §
p.Q243*	c.727C>T	PVS1, PM2_Supp, PP4	P §
p.R276Qfs*51	c.827del	PVS1, PP4, PM2_Supp	P §
p.G287V	c.860G>T	PP1_Strong, PM1_Supp, PM2_Supp, PP3, PP4, PS3_Supp	LP
p.N289K	c.867C>G	PS4_Mod, PP1, PM1_Supp, PM2_Supp, PM5_Supp, PP4, PS3_Supp	LP
p.R295C	c.883C>T	PM1, PM2_Supp, PP3, PS4_Mod, PP1_Strong, PS2/PM6_Supp, PP4, PS3_Supp	P
p.N327K	c.981C>G	BS1, BS3_Supp	VUS -> LB
p.P343S	c.1027C>T	BP4, BS3_Supp	VUS -> LB
p.S362F	c.1085C>T	BS3_Supp	VUS
**Controls**			
p.T186=	c.558A>G	BP7, BS3_Supp	VUS -> LB
p.V413=	c.1239C>T	BS1, BP7, BS3_Supp	LB
p.S148L	c.443C>T	PS4, PS2/PM6_VS, PM1, PM2_Supp, PP4, PP3, PS3_Supp	P †
p.R177*	c.529C>T	PVS1, PM2_Supp, PP4	P §

§As per ACMG-AMP, PVS1 (predicted NMD) is applied and not PS3_supp to avoid overweighting the same LoF mechanism. †The p.S148L variant was observed in individuals of the NMR cohort and was incorporated as a well-established pathogenic reference variant in the functional assays. Contribution of ACMG-AMP evidence criteria varied across variants. For protein-truncating variants, classification was primarily driven by predicted LoF and *HNF1B*-specific phenotype. In contrast, classification of several P/LP missense and in-frame deletion variants relied predominantly on genetic and clinical evidence (including segregation, where available). For variants lacking segregation data (p.V2L, p.P60R, p.N327K, p.P343S), classification was primarily supported by functional evidence in combination with population and in silico data. BS1 = benign strong. BS3_Supporting = benign supporting. BP4, BP7 = benign supporting. PVS1 = pathogenic very strong. VS = Very strong. PS2 = pathogenic strong. PS2/PM6_Supporting = pathogenic supporting. PS2/PM6_Moderate = pathogenic moderate. PS3_Supporting = pathogenic supporting. PS4 = pathogenic strong. PS4_Moderate = pathogenic moderate. PM1, PM2, PM4 = pathogenic moderate. PM1_Supporting, PM2_Supporting = pathogenic supporting. PM4_Moderate = pathogenic moderate. PP1, PP3 = pathogenic supporting. PP1_Moderate = pathogenic moderate. PP1_Strong = pathogenic strong. LP = likely pathogenic. P = pathogenic. VUS = variants of uncertain significance. LB = Likely benign. B = benign.

Supplementary Table S2. Forward and reverse primer sequences for 17 *HNF1B* variants and controls.

Variants	Forward Primer Sequence (5' - 3')	Reverse Primer Sequence (5' - 3')
p.V2L (c.4G>C)	CGATAAGGTACCCATG**C**TGTCCAAGCCACGTC	GACGTGAGCTTGGACA**G**CATGGGTACCTTATCG
p.S7Rfs*7 (c.18del)	AGACGCTGCCCCGTCCCCTGGCAG	CTGCCAGGGGACGGGGCAGCGTCT
p.G20R (c.58G>A)	CCTGCTGAGCTCC**A**GGGTCACCAAGGA	TCCTTGGTGACCC**T**GGAGCTCAGCAGG
p.L48Rfs*77 (c.143del)	AGACGCTGCCCCGGTCCCCTGGCA	CTGCCAGGGGACCGGGGCAGCGTC
p.P60R (c.179C>G)	GCCCGACACCAAGC**G**GGTCTTCCATACTC	GAGTATGGAAGACC**C**GCTTGGTGTCGGGC
p.Q131H (c.393A>T)	GGGTTACATGCAGCA**T**CACAACATCCCCCAG	CTGGGGGATGTTGTG**A**TGCTGCATGTAACCC
p.R137_K161del (c.410_484del)	AGCAACCAAACATCCCCAGACCCAGAAGCGT	AGGCTTCTGGGTCTGGGGATGTTGTTGCT
p.S148L (c.443C>T)	CACCGGCCTGAACCAGT**T**GCACCTCTC	GAGAGGTGC**A**ACTGGTTCAGGCCGGTG
p.R177* (c.544C>T)	CGACAATTCAACCAGACGGTCCAGAGTTCTGGAAA	TTTCCAGAACTCTGGACCGTCTGGTTGAATTGTCG
p.Q182* (c.544C>T)	GGGGCCCGCGTCC**T**AGCAAATCTTGTAC	GTACAAGATTTGCT**A**GGACGCGGGCCCC
p.T186= (c.558A>G)	CGACAATTCAACCAGAC**G**GT CAGAGTTCTGGAAA	TTTCCAGAACTCTGGAC**C**GTCTGGTTGAATTGTCG
p.Q243* (c.727C>T)	CCCACGGCCTGGTCTCCAACTTGGTC	GACCAAGTTGGAGACCAGGCCGTGGG
p.R276Qfs*51 (c.827del)	GGCCTGGGCTCCAAGTTGGTCACTGAG	CTCAGTGACCAACTTGGAGCCCAGGCC
p.G287V (c.860G>T)	CCCACGGCCTGG**T**CTCCAACTTGGTC	GACCAAGTTGGAG**A**CCAGGCCGTGGG
p.N289K (c.867C>G)	GGCCTGGGCTCCAA**G**TTGGTCACTGAG	CTCAGTGACCAA**C**TTGGAGCCCAGGCC
p.R295C (c.883C>T)	ACTTGGTCACTGAGGTC**T**GTGTCTACAACTGGTTT	AAACCAGTTGTAGACAC**A**GACCTCAGTGACCA AGT
p.N327K (c.981C>G)	GACTCACAGCCTGAA**G**CCTCTGCTCTCCC	GGGAGAGCAGAGG**C**TTCAGGCTGTGAGTC
p.P343S (c.1027C>T)	CAGCCCAGCTCCTCT**T**CTCCAAACAAGCTGT	ACAGCTTGTTTGGAG**A**AGAGGAGCTGGGCTG
p.S362F (c.1085C>T)	GCAGGGAAACAATGAGATCACTT**T**CTCCTCAACAA TCA	TGATTGTTGAGGAG**A**AAGTGATCTCATTGTTTCCCTGC
p.V413= (c.1239C>T)	GAGGAGGTTTGCCCCCAGT**T**AGCACCTTGA	TCAAGGTGCT**A**ACTGGGGGCAAACCTCCTC

Supplementary Table S3. Overview of ACMG-AMP criteria used in the study.

ACMG-weights	HNF1B-specific Weights	Specifications	Thresholds
		Benign criteria	
BS1	BS1	GnomAD v4.1.0	Grpmax FAF ≥ 1/30,000 (0.000033 or 0.0033%)
BS3 [¥]	BS3_Supp	Transactivation activity in HeLa cells	≥85%
BP4	BP4	AlphaMissense scores	≤ 0.34
BP7	BP7	Variant is synonymous with no splice impact, and the nucleotide is weakly/moderately conserved	
		Pathogenic criteria	
PVS1	PVS1	Variants sensitive to NMD with expected LoF	
PS4	PS4<br>PS4_Mod	Variant met PM2_Supp and observed in multiple cases	7 or more<br>4–6
PS3 [¥]	PS3_Supp	Transactivation activity in HeLa cells	≤50%
PM1 [§]	PM1	Residues in direct contact with DNA, and applicable for in-frame deletion variants within the DBD	Q136, R137, N146, Q147, S148, H149, N155, K156, K161, K164, R232, R235, K237, Q243, N255, R261, R295, N298, N302, R304, K305

	PM1_Supp	Residues in critical domains with no direct contact with DNA	DD: 1–30, POU-specific: 101–180, POU-homeo: 229–310
PM2	PM2_Supp	GnomAD v4.1.0	Grpmax FAF ≤ 1/333,000 (0.000003 or 0.0003%)
PM4	PM4_Mod	Applicable for in-frame deletion variants	(4–6 aa)
PS2	PS2/PM6_Supp	Assumed de novo and phenotype not specific	
	PS2/PM6_Mod	Assumed de novo and phenotype specific	Faguer score ≥ 8
	PS2/PM6_Very strong	Confirmed de novo and phenotype specific in two probands	Faguer score ≥ 8
PP1	PP1		1 family (3 meiosis) and > 1 family (2 meiosis)
	PP1_Mod	Phenotype cosegregates with disease	1 family (4 meiosis) and > 1 family (3 meiosis)
	PP1_Strong		1 family (5 meiosis) and > 1 family (4 meiosis)
PP3	PP3	AlphaMissense scores	≥ 0.564
PP4 *	PP4	Phenotype is consistent with *HNF1B*-disease	Faguer score ≥ 8

* According to the Faguer guidelines, MODY is characterized by a young onset of diabetes (≤ 35 years), a non-obese presentation, and no insulin dependence [1]. However, since most *HNF1B* patients are insulin-dependent, we assigned 4 points if diabetes was diagnosed before the age of 35 with absent autoantibodies. Other criteria remained unchanged unless stated. §Residues used for application of the PM1 criterion were based on a previously published study [2]. ¥ACMG-AMP PS3_Supp and BS3_supp evidence assignment was based on transactivation activity measured in HeLa cells using a rat albumin promoter-driven reporter assay. Assay performance was calibrated using 18 independently classified control variants (unpublished data), and thresholds for evidence strength were defined and supported by Odds of Pathogenicity (OddsPath) analysis, in accordance with recommendations from ClinGen Sequence Variant Interpretation Working Group and Brnich et al. [3,4]. All variants were classified based on MDEP *HNF1A*-specific ACMG-AMP variant interpretation guidelines (clinicalgenome.org, accessed 13 May 2026), adapted for the *HNF1B* gene for BS3, BP4, PS3, PM1, PS2, PP3, and PP4. Supp = supporting. Mod = moderate. BS1 = benign strong. BS3_Supporting = benign supporting. BP4 and BP7 = benign supporting. PVS1 = pathogenic very strong. PS2 = pathogenic strong. PS2/PM6_Supporting = pathogenic supporting. PS2/PM6_Moderate = pathogenic moderate. PS3_Supporting = pathogenic supporting. PS4 = pathogenic strong. PS4_Moderate = pathogenic moderate. PM1, PM2, PM4 = pathogenic moderate. PM1_Supporting, PM2_Supporting = pathogenic supporting. PM4_Moderate = pathogenic moderate. PP1, PP3 = pathogenic supporting. PP1_Moderate = pathogenic moderate. PP1_Strong = pathogenic strong.

References

1. Faguer, S.; Chassaing, N.; Bandin, F.; Prouheze, C.; Garnier, A.; Casemayou, A.; Huart, A.; Schanstra, J.P.; Calvas, P.; Decramer, S.; et al. The HNF1B score is a simple tool to select patients for HNF1B gene analysis. *Kidney Int.* **2014**, *86*, 1007–1015. https://doi.org/10.1038/ki.2014.202.
2. Lu, P.; Rha, G.B.; Chi, Y.-I. Structural Basis of Disease-Causing Mutations in Hepatocyte Nuclear Factor 1β. *Biochemistry* **2007**, *46*, 12071–12080. https://doi.org/10.1021/bi7010527.
3. ClinGen Consortium. The Clinical Genome Resource (ClinGen): Advancing genomic knowledge through global curation. *Genet. Med.* **2025**, *27*, 101228.
4. Brnich, S.E.; Tayoun, A.N.A.; Couch, F.J.; Cutting, G.R.; Greenblatt, M.S.; Heinen, C.D.; Kanavy, D.M.; Luo, X.; McNulty, S.M.; Starita, L.M.; et al. Recommendations for application of the functional evidence PS3/BS3 criterion using the ACMG/AMP sequence variant interpretation framework. *Genome Med.* **2019**, *12*, 3. https://doi.org/10.1186/s13073-019-0690-2.
